# Early discharge predictors among inpatient crack cocaine users

**DOI:** 10.47626/2237-6089-2021-0401

**Published:** 2023-05-19

**Authors:** Edgar Klein, Felipe Ornell, Vinícius S. Roglio, Juliana N. Scherer, Anne O. Sordi, Jaqueline B. Schuch, Felix H. P. Kessler, Lisia von Diemen

**Affiliations:** 1 Centro de Pesquisa em Álcool e Drogas Hospital de Clínicas de Porto Alegre Universidade Federal do Rio Grande do Sul Porto Alegre RS Brazil Centro de Pesquisa em Álcool e Drogas, Hospital de Clínicas de Porto Alegre, Universidade Federal do Rio Grande do Sul, Porto Alegre, RS, Brazil.; 2 Programa de Pós-Graduação em Psiquiatria e Ciências do Comportamento Universidade Federal do Rio Grande do Sul Porto Alegre RS Brazil Programa de Pós-Graduação em Psiquiatria e Ciências do Comportamento, Universidade Federal do Rio Grande do Sul, Porto Alegre, RS, Brazil.

**Keywords:** Treatment retention, drug addiction, substance use disorder, cocaine use disorder, crack dependence

## Abstract

**Introduction:**

High rates of early hospital discharge are often observed in crack cocaine users and are related to adverse outcomes and increased public spending. This study evaluated clinical and sociodemographic factors associated with early treatment discharge among crack users.

**Methods:**

The sample comprised 308 men diagnosed with crack cocaine use disorder (crack only), aged 18 to 65 years, admitted between 2013 and 2017 to a male-only hospital unit to treat substance use disorders. Sociodemographic and clinical data were obtained using the Addiction Severity Index, 6th version, and a Sociodemographic Questionnaire.

**Results:**

Early discharge (within 7 days) was significantly associated with lack of own income, insufficient family support, being single, and recent homelessness. Regarding drug use, lower treatment retention was related to younger age of crack use onset, recent alcohol use, and nicotine use. Factors such as age, skin color, and educational level showed no relation to the outcome.

**Conclusion:**

Our findings suggest that presence of characteristics verifiable at the time of admission may be related to crack users’ treatment retention. Identification of these factors can contribute to target interventions in order to improve treatment adherence in crack cocaine users.

## Introduction

Epidemiological studies have shown increased crack consumption (cocaine smoking) in Brazil over recent years.^1,2^ Research estimates that nearly 0.7% of the adult population and 35% of illicit drug users living in state capitals regularly smoke crack.^1^ Crack dependence is related to higher mortality rates due to violence, to higher incarceration rates, and to behaviors that increase the occurrence of negative health outcomes, such as risk behaviors for human immunodeficiency virus (HIV) and viral hepatitis contamination^3-7^ and attempted suicide.^8^ In a 12-year follow-up study with crack users, Dias et al. observed high mortality (20%) and incarceration (10%) rates.^9^ Studies have also observed violent behaviors and involvement in crime in these users.^7,9-12^

Although the prevalence of crack use is lower than that of other drugs, such as tobacco, alcohol, and cannabis, this substance is responsible for most psychiatric hospitalizations in Brazil.^6,10,12,13^ Detoxification hospitalization is an important element of treatment; besides helping manage initial withdrawal symptoms, the stabilization stage enables motivational strategies to be developed, which can improve therapeutic adherence.^14,15^ Nevertheless, individuals with crack dependence have high rates of early discharge requests in voluntary hospitalizations in detoxification units.^16-18^ Previous studies indicate that the rate of early withdrawal after psychiatric hospitalization ranges from 3 to 82%, with the highest percentages found among drug addicts.^16,19^ This abandonment is related to higher readmission rates,^20,21^ and higher mortality rates from suicide and violence.^16,17,22^ A recent Canadian study observed that the readmission rate among illicit drug users discharged early (within 7 days of hospitalization or less) is 50% in one year.^23^

Studies conducted in North America and Europe identified several factors associated with low retention during detoxification hospitalization in drug users, such as number of previous admissions, unfavorable employment status,^20^ involvement in crime,^20,24,25^ being intoxicated at the time of admission, being a racial minority in the hospitalization unit, being younger, being male,^24^ and early drug use onset.^26^ Factors related to greater social vulnerability and greater severity of dependence such as irregular housing or homelessness, disrupted social ties, and lack of access to social care services were also associated with this outcome.^18,27^

Although previous studies have investigated factors related to treatment retention, no Brazilian studies have addressed this issue in the social, cultural, and clinical context of crack users. Unveiling the predictive factors of early discharge can help to identify individuals more susceptible to this outcome and those who require personalized interventions to develop more effective treatment strategies. Pertinent information that can be easily assessed at the time of admission may be an essential factor concerning treatment retention. As such, this study aimed to assess which social, demographic, and clinical factors, verifiable at the time of admission, may be related to low treatment retention in male crack users.

## Methods

### Design and sample

This cross-sectional study was approved by the Research Ethics Committee at the Hospital de Clínicas de Porto Alegre (HCPA, no. 2018-0487) and was carried out in accordance with the Declaration of Helsinki. All subjects signed informed consent before enrollment on the study. The sample comprised 308 individuals diagnosed with cocaine use disorder (cocaine smoking/crack) consecutively recruited at a hospitalization unit specialized in treatment of drug addiction – the Unidade Álvaro Alvim (HCPA) in Rio Grande do Sul, Brazil – from 2013 to 2017. This detoxification unit only admits male inpatients.

Inclusion criteria were being male, being between 18 and 65 years old, having a diagnosis of cocaine use disorder (cocaine smoking/crack) according to the Diagnostic and Statistical Manual of Mental Disorders, fourth version (DSM-IV) criteria, and having cognitive ability to understand the instruments applied and authorize access to medical record data. Users of inhaled cocaine and participants who had not answered the instruments by discharge were excluded.

Undergraduate students received training to apply the protocol and were supervised by a senior researcher (psychologist or psychiatrist). After signing informed consent, subjects answered the research protocol, which took place between the second and fifth day of hospitalization. Data were reviewed to ensure better quality. The research protocol included a sociodemographic questionnaire covering personal and family income, schooling, employment, housing, and marital status. Subjects also answered the Addiction Severity Index, 6th version (ASI-6),^28^ which evaluates the impact of licit and illicit substance use on patients’ lives in several different areas: medical, occupational status, legal status, family/social relationships, psychiatric, alcohol use, and use of other drugs.

### Statistical analysis

The main outcome was the length of hospital stay, defined by the time, in days, that subjects remained in treatment at the hospital. Besides medical hospital discharge, after stabilization of the patient’s clinical condition, discharge could be requested by patients themselves or could be recommended by the hospital staff (usually for administrative reasons). Length of hospital stay was divided into quartiles (minimum 1 day – maximum 76 days). The first quartile was equivalent to 7 days of hospitalization or less and considered the early discharge group. To identify predictors of early discharge, we compared this group with the group of subjects that remained 8 days or more hospitalized in the detoxification unit. We selected possible predictors taking into account findings from previous studies conducted in other countries that had evaluated factors related to early discharge of drug users.^18,20,24-27^

Quantitative variables with normal or symmetric distribution were expressed as mean and standard deviation and compared between groups using the *t* test for independent samples. Asymmetric quantitative variables were expressed as median and interquartile interval and compared using the Mann-Whitney test. Categorical variables were expressed as absolute and relative frequencies and underwent the chi-square test of association. All analyses were performed on IBM SPSS® version 18 software, using a 95% confidence level.

## Results

Mean hospital stay was 18.7±14.8 days. Early discharge, which occurred within 7 days of admission or less, accounted for 27% of the hospitalizations evaluated (n = 83). Table 1 presents the analysis of sociodemographic, family, employment, housing, and legal status data stratified by length of hospital stay. Age, skin color, schooling and employment situation were similar between the lowest (≤ 7 days of hospitalization) and highest retention groups. Shorter hospital stay was associated with insufficient income (p = 0.027), being single (p = 0.007), having no children (p = 0.011), and being homeless/staying in shelters in the past six months (p = 0.044).

**Table 1:** Sociodemographic, family, employment, housing, and legal status data by length of hospital stay (in days)

	Total	Length of hospital stay		

		≤ 7 days (n = 83)	≥ 8 days (n = 225)	p-value
Age (years)*	33.5±8.2	32.2±8.1	34±8.2	0.088
Skin color^†^				0.615
White	148 (48.5)	38 (45.8)	110 (49.5)	
Black	74 (24.3)	19 (22.9)	55 (24.8)	
Other	83 (27.2)	26 (31.3)	57 (25.7)	
Schooling^†^				0.912
None	60 (19.5)	17 (20.5)	43 (19.5)	
Up to 8 years	153 (49.7)	42 (50.6)	111 (49.3)	
Between 9 and 11 years	84 (27.3)	22 (26.5)	62 (27.6)	
More than 12 years	11 (3.6)	2 (2.4)	9 (4)	
Partner status^†^				**0.007**
Single	249 (81.4)	77 (92.8)^‡^	172 (77.1)^‡^	
Married/living together	41 (13.4)	4 (4.8)^‡^	37 (16.6)^‡^	
Widowed/divorced	16 (5.2)	2 (2.4)	14 (6.3)	
Children (yes)^†^	195 (63.3)	43 (51.8)	152 (67.6)	**0.011**
Number of dependents who need regular financial support^†^				0.264
None	138 (44.8)	37 (44.6)	101 (44.9)	
1 or 2	105 (34.1)	33 (39.8)	72 (34.1)	
3 or more	65 (21.1)	13 (15.7)	52 (23.1)	
Employment^†^				0.120
Full-time	107 (35.1)	23 (27.7)	84 (37.8)	
Half-time	74 (24.3)	20 (24.1)	54 (24.3)	
Looking for a job	75 (24.6)	28 (33.7)	47 (21.2)	
Unemployed	49 (16.1)	12 (14.5)	37 (16.7)	
Has enough income to pay for basic needs of his dependents^†^	147 (47.7)	31 (37.3)	116 (51.6)	**0.027**
Lived on the street or in shelters (last 6 months)^†^	95 (31)	33 (39.8)	62 (27.8)	**0.044**
Ever been arrested (yes)^†^	124 (41.5)	34 (45.3)	90 (40.2)	0.499

Data expressed as * mean ± standard deviation, *t* test for independent samples; or ^†^ absolute frequency (%), chi-square test of association.

^‡^ Statistical significance.

The results show that factors related to the presence or quality of interpersonal relationships were also associated with the outcome (Table 2), including spending time with family members or close friends (p = 0.044) and talking to people close to them about their feelings and problems (p = 0.049). Hospitalizations that occurred in the colder months (June, July, August, and September) showed a tendency to longer duration (p = 0.060). Difficulty in controlling temperament also seems to be related to shorter hospital stays (p = 0.060). Variables related to legal status and/or criminal activities, religious activities, and having HIV showed no significant differences between groups.

**Table 2:** Interpersonal relationships and personal characteristics, by length of hospital stay (in days)

	Total	Length of hospital stay		

		≤ 7 days (n = 83)	≥ 8 days (n = 225)	p-value
Had a romantic/sexual relationship with a partner (last month)	193 (65)	49 (67.1)	144 (64.3)	0.659
Told people close to you about your feelings or problems (last month)	128 (67.7)	27 (56.3)	101 (71.6)	**0.049**
Spent time with partner(s) (last month)	167 (87.4)	41 (85.4)	126 (88.1)	0.626
Spent time with family or close friends (last month)	238 (80.7)	53 (72.6)	185 (83.3)	**0.044**
Difficulty in controlling temperament	109 (38.2)	33 (47.8)	76 (35.2)	0.060
Referral to treatment				0.157
By yourself, spouse, family member, or friend	217 (71.9)	54 (65.9)	163 (74.1)	
By institution, health professional, or judge	85 (28.1)	28 (34.1)	57 (25.9)	
Hospitalization at times of the year				0.060
Cold season (June to September)	179 (58.1)	41 (49.4)	138 (61.3)	
Warm season (October to May)	129 (41.9)	42 (50.6)	87 (38.7)	
Religious activity	135 (45.9)	32 (44.4)	103 (46.4)	0.773
Sexual abuse	34 (11.9)	8 (11.8)	26 (11.9)	0.971
HIV/AIDS	31 (10.2)	7 (8.5)	24 (10.8)	0.568

Data expressed as absolute frequency (%), chi-square test of association.

Table 3 shows the relationship between length of hospital stay and drug use profile. Individuals with shorter stays had an earlier age of crack use onset (p = 0.011). There was no difference in age of onset use of other licit and illicit substances between groups. Although we found a high prevalence of positive urine test for cocaine at the time of admission (89.5% of participants), this variable was not a predictor of early discharge. On the other hand, alcohol use in the days prior to hospitalization was significantly different between the two groups. Individuals discharged within 7 days or fewer reported last alcohol consumption between 3 and 8 days prior to admission, while those with longer hospital stays reported last consumption between 5 and 15 days (p = 0.02). Prevalence of smoking was 81% in the total sample, being more prevalent in the early discharge group (90%) compared to the group with longer hospital stay (78%, p = 0.033). The early discharge group had younger age at first treatment for any substance use disorder by 3.6 years (p = 0.005) compared with the longer hospital stay group.

**Table 3:** History and pattern of drug use by length of hospital stay (in days)

	Total	Length of hospital stay		

		≤ 7 days (n = 83)	≥ 8 days (n = 225)	p-value
Age at first use of tobacco*	14.3±4.3	14.36±3.1	14.3±4.7	0.957
Age at first use of alcohol*	14.5±3.6	14.7±3.3	14.4±3.7	0.597
Age at first use of marijuana*	15.1±4.4	14.8±3.4	15.2±4.7	0.439
Age at first use of cocaine*	18±4.6	17.7±4.4	18.1±4.7	0.565
Age at first use of crack*	24.2±8	22.2±6.2	24.9±8.5	**0.011**
Urine test strip for cocaine^†^				0.671
Positive	197 (89.5)	60 (88.2)	137 (90.1)	
Negative	23 (10.5)	8 (11.8)	15 (9.9)	
Recent crack use (last 30 days)^**§**^	20 [5-30]	15 [4.5-30]	20 [5-30]	0.679
How many days ago did you last use alcohol^**§**^	7 [4-14]	6 [3-8]	9 [5-15]	**0.002**
Current smoking^†^	216 (81.5)	63 (90)	153 (78.5)	**0.033**
Age at first drug treatment*	26.1±8.5	23.5±7	27.1±8.8	**0.005**

Data expressed as * mean ± standard deviation, *t* test for independent samples; or ^†^ absolute frequency (%), chi-square test of association; or ^§^ median [Q1-Q3], Mann-Whitney test.

## Discussion

Our study showed that sociodemographic data verifiable at the time of admission can help recognize a patient profile with a greater chance of treatment retention. Patients who are married, have children, a family and social support network, no history of homelessness, an income that allows them to pay for basic needs, and who started using crack later in life stayed in hospital longer, indicating that this profile may benefit more from this type of voluntary hospitalization. Patients without these characteristics may need a specific treatment protocol to improve treatment retention.

Our results corroborate those found by previous studies, in which low treatment retention was associated with absence of family ties, such as spouse and children.^7,23^ In a sample with characteristics similar to ours, hospitalized patients reported that a lack of social support is an important factor related to motivating treatment. Deficient family and social support have been associated with greater vulnerability and severity of substance use disorders, related as both its cause and consequence.^4,27^ Being single or having disrupted social bonds and lacking interpersonal relationships that allow space for sharing feelings or for spending time together, are predictors of early discharge in our study and confirm the role of these vulnerabilities in treatment retention.^29,30^ The association between having no children and the outcome studied should be interpreted with caution as it may be related to marital status, or it may reveal a reason for less motivation for treatment. Reestablishing or promoting new social bonds in these individuals should then be considered a key strategy in developing therapeutic plans, thus positively interfering in treatment retention, as already suggested in previous studies.^7^

Factors related to socioeconomic vulnerability were also associated with a shorter hospital stay. Unfavorable employment situations and income informality are also vulnerabilities associated with severity of substance use disorder.^4,7^ Among our findings, one of the main variables related to early discharge was lacking income for their basic needs and that of their dependents, an association already described in previous studies conducted with substance users.^1,31,32^ Homelessness or staying in shelters in the last 6 months may also contribute to greater vulnerability and severity of substance use disorder,^4,32^ as well as being a consequence of previously mentioned issues such as disruption of personal ties and absence or insufficiency of income.^1,2^ The observed trend of longer hospital stays in cold months may be more related, in some cases, to the unfavorable housing situation rather than to authentic motivation for treatment. We found no other studies that evaluated this specific factor with respect to the outcome days of hospitalization in populations of substance users.

Information on the history and profile of substance use also provides important data regarding retention on a detoxification treatment, indicating a need to adapt the initial therapeutic plan for specific cases.^16,33^ In this context, greater attention seems to be needed for individuals with earlier onset of crack use, who sought first treatment earlier (possibly due to severity), and who reported more recent alcohol consumption, which may result in symptoms of additional craving or withdrawal.

Together, these data show that lower treatment retention is observed in users with longer addiction time, greater impairment of executive functions, greater severity and chronicity of substance use disorder, and greater difficulty in coping with withdrawal symptoms, corroborating previous studies.^34^ While we found no difference regarding frequency of alcohol use, recent consumption is a risk factor and may indicate greater neurological and executive function impairment caused by the concomitant use of these two substances.^35,36^ The high smoking rate found in individuals with lower treatment retention underscores the importance of establishing strategies for craving management and pharmacological control of nicotine withdrawal symptoms as early as possible.^35,37,38^

The high rate of hospital discharge within seven days or less, observed in over one quarter of admissions in the sample studied, calls attention to the need for a specific approach to this group of patients, aiming to optimize this therapeutic resource. Research suggests that initial abstinence is associated with stabilization of several physiological and brain functions related to the craving and relapse processes. Several studies highlight strategies for preventing early discharges that are effective for improving treatment efficacy.^1,7,16,32,37-41^ Identifying the factors potentially related to these discharges can help to better predict length of hospital stay and, consequently, can help with making the most appropriate therapeutic decisions that will enhance the effectiveness of the unique therapeutic plan, as well as with choosing the most appropriate environment for it.

However, this study has some limitations. First, the hospitalizations analyzed took place in only one hospital, over a specific period of time, and were consequent to a specific treatment plan, thus our results may not be generalizable to other medical environments or therapeutic strategies. Second, our findings do not discriminate the reasons for early discharges, only their association with the variables analyzed. Therefore, we do not differentiate between discharges by patient request or decision or discharges for administrative reasons. Very early discharges (usually between one and three days) may have occurred before there was time to administer the instruments and so unfortunately data from such patients were not included in our analyses. From 2013 to 2017, there were 499 admissions of crack to the hospital unit under study, but only 308 patients met the sample inclusion criteria and agreed to participate in the study. Finally, considering the nature of bivariate analyses, some associations may be related to confounding factors.

## Conclusions

Our findings suggest the existence of characteristics verifiable at the time of admission that may be related to crack users’ treatment retention. Early identification of these factors may allow for optimization of therapeutic strategies. Decisions as to which setting, outpatient or hospital, is more appropriate for detoxification treatment can be based on these characteristics. Regarding admission to specialized hospitals, our results identify intervention priorities to improve retention and decrease early discharge rates.
